# Association of Remimazolam-Based Versus Desflurane-Based Maintenance with Early Gastrointestinal Recovery After Laparoscopic Cholecystectomy: A Single-Center Retrospective Cohort Study

**DOI:** 10.3390/jcm15114202

**Published:** 2026-05-29

**Authors:** Byeong Gwan Noh, Eun Ji Park, Myunghee Yoon, Hyung Il Seo, Young Mok Park, Myeong Hun Oh, Hyeon-Jeong Lee, Jeong-Min Hong, Boo-young Hwang, Unji Kim, Mingyu Kim

**Affiliations:** 1 Department of Surgery, Biomedical Research Institute, Pusan National University Hospital, Busan 49241, Republic of Koreaseohi71@hanmail.net (H.I.S.);; 2 Department of Surgery, School of Medicine, Pusan National University, Yangsan 50612, Republic of Korea; 3 Department of Anesthesia and Pain Medicine, Biomedical Research Institute, Pusan National University Hospital, Busan 49241, Republic of Koreaccarrot@pusan.ac.kr (J.-M.H.);; 4 Department of Anesthesia and Pain Medicine, School of Medicine, Pusan National University, Yangsan 50612, Republic of Korea

**Keywords:** cholecystectomy, laparoscopic, remimazolam, gastrointestinal motility, postoperative period, perioperative care

## Abstract

**Background/Objectives:** In standardized low-event surgery such as laparoscopic cholecystectomy (LC), discharge-based outcomes may be insufficiently sensitive to capture differences in recovery trajectory. We investigated whether early gastrointestinal recovery after standardized LC differed according to anesthetic maintenance strategy. **Methods:** This single-center retrospective cohort study included consecutive adults who underwent scheduled LC between September 2023 and December 2025 within a standardized perioperative pathway. The primary exposure was anesthetic maintenance strategy, comparing remimazolam-based with desflurane-based maintenance. The primary outcome was time to first flatus. Key secondary outcomes included postoperative day 1 (POD 1) high-sensitivity C-reactive protein (hs-CRP), C-reactive protein-to-albumin ratio (CAR), diet delay, prolonged hospital stay, postoperative nausea and vomiting, and 30-day readmission. Associations were evaluated using a log-normal accelerated failure time model, multivariable logistic regression, and log-transformed linear models for inflammatory markers. **Results:** A total of 316 patients were included (remimazolam, *n* = 171; desflurane, *n* = 145). Time to first flatus was shorter in the remimazolam group, with an unadjusted median difference of 8 h (28.0 [24.0–37.0] vs. 36.0 [28.0–52.0] h). After adjustment, remimazolam-based maintenance remained associated with a 21% shorter time to first flatus (time ratio, 0.79; 95% confidence interval [CI], 0.72–0.86; *p* < 0.001), corresponding to an adjusted median reduction of 8.0 h. The remimazolam group also showed earlier flatus recovery across predefined time windows and lower POD 1 hs-CRP and CAR, whereas later outcomes were largely similar. **Conclusions:** In standardized LC, early gastrointestinal recovery appeared more sensitive to anesthetic maintenance strategy than discharge-based outcomes. These findings support the use of early functional recovery measures, in addition to discharge timing, when evaluating perioperative recovery in low-event short-stay surgery.

## 1. Introduction

Laparoscopic cholecystectomy (LC) is one of the most standardized minimally invasive abdominal procedures and remains the standard treatment for most benign gallbladder diseases [1,2]. Because major postoperative complications are uncommon and recovery is typically rapid, LC is increasingly managed within short-stay or ambulatory pathways [3,4]. In this setting, conventional postoperative endpoints such as length of stay, readmission, or overt complications often show limited variability and may be insufficiently sensitive to capture clinically meaningful differences in recovery quality [5,6,7].

This limitation is particularly relevant when perioperative care is delivered within a structured pathway. Standardized diet advancement and discharge routines can compress downstream outcome variability, even when the pace of physiologic recovery differs between patients [5,6,7]. As a result, recovery after LC may be more informatively evaluated by the trajectory of early functional recovery rather than by discharge-based outcomes alone.

Among the domains of postoperative recovery, gastrointestinal function is especially relevant after abdominal surgery because it is closely linked to oral intake progression and overall postoperative flow [8]. In a short-stay LC pathway, return of bowel function is not merely a time stamp but a practical marker of early physiologic recovery. Accordingly, time to first flatus provides a pragmatic and clinically meaningful endpoint for evaluating early gastrointestinal recovery in routine perioperative practice [8].

Anesthetic maintenance strategy is a modifiable perioperative factor that may influence early postoperative recovery, but its relevance may be underestimated when assessed only through downstream discharge-based outcomes [5,6,7]. We therefore examined whether early gastrointestinal recovery after standardized laparoscopic cholecystectomy differed according to anesthetic maintenance strategy, using time to first flatus as a pragmatic marker of early recovery. We hypothesized that maintenance strategy would be associated with differences in the timing of early gastrointestinal recovery within this standardized short-stay pathway.

## 2. Materials and Methods

### 2.1. Study Design and Study Population

This single-center retrospective cohort study was conducted at a tertiary academic hospital and included consecutive adult patients who underwent scheduled laparoscopic cholecystectomy between September 2023 and December 2025. Acute cholecystitis in this study referred to histopathologically confirmed acute cholecystitis among patients who underwent scheduled laparoscopic cholecystectomy, rather than emergency surgery for clinically diagnosed acute cholecystitis. All procedures were performed by a single experienced surgeon. Patients were excluded if they underwent conversion to open surgery, concomitant surgical procedures during the same anesthetic session, had preexisting gastrointestinal motility disorders, or had incomplete perioperative records. Patients who received mixed anesthetic regimens were also excluded to minimize exposure misclassification.

### 2.2. Perioperative Management and Anesthetic Strategy

Laparoscopic cholecystectomy was performed using a standardized technique with carbon dioxide pneumoperitoneum. Perioperative care, including postoperative dietary advancement and discharge planning, followed a structured institutional protocol applied consistently throughout the study period. Under this pathway, clinically stable patients were scheduled to begin diet advancement on postoperative day 1 (POD 1), and discharge was generally considered on POD2–3 once oral intake and overall clinical stability were confirmed. General anesthesia was administered in all patients. Anesthetic care was delivered or supervised by a single attending anesthesiologist throughout the study period. Intraoperative analgesia was provided with remifentanil infusion according to institutional practice. Neuromuscular blockade was achieved with rocuronium under quantitative neuromuscular monitoring and was routinely reversed with sugammadex guided by train-of-four monitoring. Postoperative analgesic and antiemetic management followed institutional protocols that were applied uniformly to both anesthetic groups.

The primary exposure was anesthetic maintenance strategy as delivered in routine clinical practice, and the comparison was intended to reflect real-world anesthetic strategies rather than the isolated pharmacologic effect of a single agent. This was not a randomized comparison, and no formal patient-level allocation protocol was used to assign patients to remimazolam-based or desflurane-based maintenance. During the study period, the choice of maintenance strategy largely reflected the usual practice pattern of the single attending anesthesiologist, rather than predefined selection criteria based on measured patient characteristics. Remimazolam-based anesthesia was defined as induction and maintenance with remimazolam in combination with remifentanil, reflecting a balanced total intravenous anesthesia technique. Desflurane-based anesthesia was defined as induction with an intravenous propofol bolus followed by maintenance with desflurane in combination with remifentanil, reflecting a balanced inhalational anesthesia technique. Anesthesia duration and the mean weight-normalized intraoperative remifentanil infusion rate were recorded as descriptors of intraoperative anesthetic management and were additionally evaluated in robustness analyses.

### 2.3. Study Outcomes

The primary outcome was time to first flatus, defined as the interval in hours from the end of anesthesia to the first documented passage of flatus. Time to first flatus was selected as a pragmatic marker of early postoperative gastrointestinal functional recovery. Passage of flatus was assessed repeatedly in nursing documentation during the inpatient stay as part of routine postoperative diet advancement decisions, and flatus time was available for all patients included in the primary analysis. Key secondary outcomes were postoperative inflammatory burden on POD 1, protocol-defined diet delay, prolonged hospital stay, postoperative nausea and vomiting (PONV), and 30-day readmission. POD 1 inflammatory burden was assessed using high-sensitivity C-reactive protein (hs-CRP) and the C-reactive protein-to-albumin ratio (CAR), both measured on POD 1. CAR was calculated as the ratio of POD 1 hs-CRP to POD 1 serum albumin. Protocol-defined diet delay was defined as failure to initiate or advance oral intake as scheduled on POD 1 within the institutional pathway. Prolonged hospital stay was defined as a length of stay of 4 days or longer, representing deviation from the usual POD2–3 discharge target of the institutional short-stay pathway. To facilitate clinical interpretation of the primary time-to-event analysis, predefined early recovery windows of ≤24, ≤30, and ≤36 h to first flatus were also evaluated.

In exploratory analyses, we evaluated whether delayed time to first flatus was associated with downstream recovery outcomes, including protocol-defined diet delay and prolonged hospital stay. Restricted cubic spline modeling was additionally used to examine the adjusted probability of prolonged hospital stay across the range of time to first flatus. These analyses were considered exploratory and intended to assess whether variation in early gastrointestinal recovery was associated with subsequent pathway deviation.

### 2.4. Covariates

The primary adjusted model incorporated prespecified clinical covariates selected on clinical grounds: age (per 10-year increase), modified Charlson Comorbidity Index (mCCI) ≥ 3, acute cholecystitis (yes/no), history of previous abdominal surgery, and preoperative biliary intervention (endoscopic retrograde cholangiopancreatography and/or percutaneous transhepatic gallbladder drainage). An mCCI cutoff of ≥3 was selected a priori to identify patients with a higher comorbidity burden while preserving model parsimony and avoiding sparse strata in the multivariable models. Because ASA physical status may overlap conceptually with comorbidity burden, mCCI was prioritized in the primary adjustment set. This primary covariate set was intentionally parsimonious and focused on clinically relevant patient- and disease-related factors to reduce overadjustment and to avoid incorporating intraoperative variables that might lie on the pathway between anesthetic strategy and recovery outcomes.

In prespecified robustness analyses, we additionally evaluated models incorporating surgery duration, anesthesia duration, and remifentanil infusion rate. For inflammatory marker models, preoperative hs-CRP was additionally included to account for baseline inflammatory status.

### 2.5. Statistical Analysis

Continuous variables are presented as mean ± standard deviation or median (interquartile range), as appropriate, and categorical variables as number (percentage). Between-group comparisons were performed using Student’s t test or Mann–Whitney U test for continuous variables and the chi-square test or Fisher’s exact test for categorical variables, as appropriate. No formal sample-size calculation was performed because this was a retrospective cohort study including all eligible consecutive patients during the study period.

Because time to first flatus showed a right-skewed distribution, the primary analysis used a log-normal accelerated failure time (AFT) model. An AFT framework was chosen because the study focused on differences in recovery time rather than on hazard-based relative event rates. Time ratios (TRs) with 95% confidence intervals (CIs) were estimated, with TR <1 indicating a shorter time to first flatus in the remimazolam group. An unadjusted AFT model including anesthetic group alone was first fitted, followed by the primary adjusted model incorporating prespecified clinical covariates. Adjusted median times to first flatus for each anesthetic strategy were estimated from the fitted model by marginal standardization over the observed covariate distribution, and the adjusted absolute difference in hours was derived from these model-based predictions. Predefined early recovery windows (≤24, ≤30, and ≤36 h) were additionally analyzed using logistic regression with the same primary adjustment set.

POD 1 hs-CRP and CAR were analyzed after logarithmic transformation because of right-skewness; because a small number of values were zero, 0.01 was added before transformation. Exponentiated coefficients from these models are presented as geometric mean ratios. These models were adjusted for age, mCCI ≥ 3, acute cholecystitis, previous abdominal surgery, preoperative biliary intervention, and preoperative hs-CRP. Protocol-defined diet delay and prolonged hospital stay were analyzed using logistic regression and are presented as odds ratios (ORs) with 95% CIs. PONV was compared between groups using Fisher’s exact test, whereas 30-day readmission was summarized descriptively because events were infrequent.

In exploratory analyses, the association of time to first flatus with diet delay and prolonged hospital stay was assessed using logistic regression models. To improve interpretability, time to first flatus was modeled on the log scale, and effect estimates were expressed as odds ratios per 10% increase in flatus time. Restricted cubic spline modeling was used to examine the adjusted probability of prolonged hospital stay over the range of time to first flatus.

Prespecified robustness analyses examined whether the main findings were materially altered after additional adjustment for surgery duration, anesthesia duration, and remifentanil infusion rate. Additional sensitivity analyses were performed to account for possible temporal practice variation by including calendar-quarter fixed effects. For the primary outcome, further sensitivity models combined calendar-quarter fixed effects with surgery duration or anesthesia duration. Analyses were performed on a complete-case basis, and no imputation was used for missing data. Missing data were limited for primary clinical variables; inflammatory marker analyses were restricted to patients with available preoperative and POD 1 laboratory measurements. Because secondary and exploratory analyses were not adjusted for multiplicity, these findings were interpreted cautiously and primarily as supportive and hypothesis-generating. A two-sided *p* value < 0.05 was considered statistically significant. All analyses were performed using R version 4.3.1 (R Foundation for Statistical Computing, Vienna, Austria). The study is reported in accordance with the Strengthening the Reporting of Observational Studies in Epidemiology (STROBE) guidelines.

## 3. Results

### 3.1. Baseline and Perioperative Characteristics

A total of 316 patients were included in the primary analysis, comprising 171 who received remimazolam-based maintenance and 145 who received desflurane-based maintenance. Baseline and perioperative characteristics are summarized in Table 1. The remimazolam group was older than the desflurane group (63.0 [53.5–71.5] vs. 60.0 [48.0–69.0] years, *p* = 0.033) and had a higher proportion of patients with ASA physical status ≥ III (42.7% vs. 31.7%, *p* = 0.048) and modified CCI ≥ 3 (22.2% vs. 9.0%, *p* = 0.002). Sex distribution, body mass index, previous abdominal surgery, acute cholecystitis, preoperative ERCP/PTGBD, and preoperative hs-CRP were similar between groups. Operative exposure also differed between groups. Surgery duration (30 [25–45] vs. 45 [35–60] min, *p* < 0.001) and anesthesia duration (50 [45–65] vs. 60 [50–80] min, *p* < 0.001) were shorter in the remimazolam group, whereas remifentanil infusion rate was higher (0.108 [0.091–0.126] vs. 0.089 [0.075–0.101] μg/kg/min, *p* < 0.001). These baseline and perioperative differences were considered in the adjusted and sensitivity analyses.

### 3.2. Time to First Flatus

**Figure 1:**
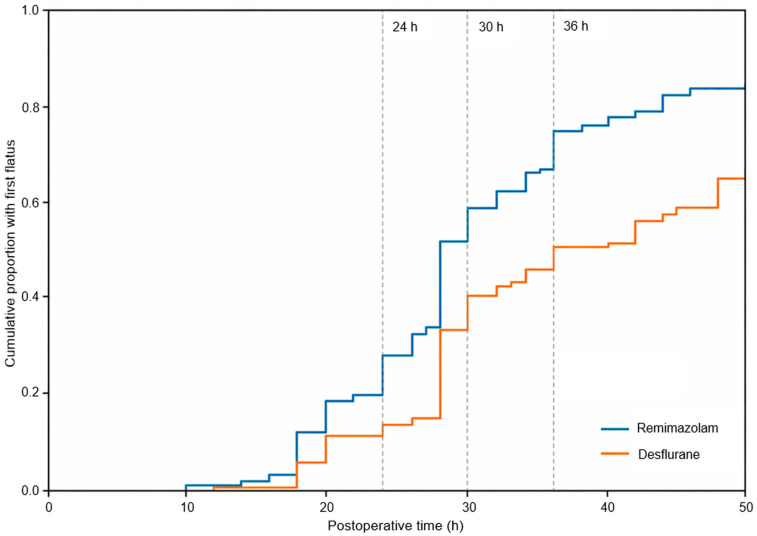
Early gastrointestinal recovery according to anesthetic maintenance strategy. Empirical cumulative proportion of patients achieving first flatus during the first 50 postoperative hours after laparoscopic cholecystectomy according to anesthetic maintenance strategy. Patients receiving remimazolam-based maintenance showed earlier gastrointestinal recovery than those receiving desflurane-based maintenance. Dashed vertical lines indicate postoperative 24, 30, and 36 h. Remimazolam, *n* = 171; desflurane, *n* = 145. The *x*-axis was truncated at 50 h for visualization.

**Figure 2:**
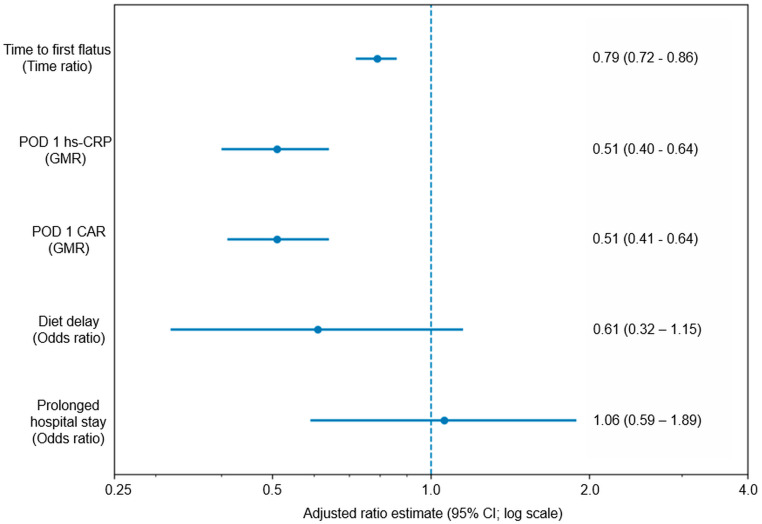
Adjusted associations of remimazolam-based maintenance with early gastrointestinal recovery, postoperative inflammatory burden, and later postoperative outcomes. Forest plot of adjusted ratio estimates comparing remimazolam-based maintenance with desflurane-based maintenance. Time to first flatus is presented as a time ratio, postoperative day 1 hs-CRP and CAR as geometric mean ratios, and diet delay and prolonged hospital stay as odds ratios. All estimates are ratio-based measures with a common null value of 1, indicated by the dashed vertical line. The *x*-axis is displayed on a logarithmic scale. POD 1 inflammatory marker analyses included patients with available preoperative and POD 1 laboratory measurements.

Time to first flatus was shorter in the remimazolam group than in the desflurane group, with an unadjusted median difference of 8 h (28.0 [24.0–37.0] h vs. 36.0 [28.0–52.0] h) (Table 2, Figure 1). After adjustment for age, modified CCI ≥ 3, acute cholecystitis, previous abdominal surgery, and preoperative ERCP/PTGBD, remimazolam-based maintenance remained associated with a 21% shorter time to first flatus (time ratio [TR], 0.79; 95% confidence interval [CI], 0.72–0.86; *p* < 0.001), corresponding to an adjusted median reduction of approximately 8.0 h based on model-derived predicted median times (30.3 h vs. 38.3 h) (Table 2, Figure 2). Full coefficients from the multivariable models incorporating surgery duration are provided in Supplementary Table S1.

### 3.3. Early Flatus Recovery Within Predefined Time Windows

Threshold-based analyses supported this pattern. The proportion of patients achieving first flatus within 24 h was higher in the remimazolam group (27.5% vs. 13.1%) and remained significant after adjustment (odds ratio [OR], 2.42; 95% CI, 1.33–4.41; *p* = 0.004). Similarly, first flatus within 30 h (58.5% vs. 40.0%; adjusted OR, 2.47; 95% CI, 1.53–3.99; *p* < 0.001) and within 36 h (74.9% vs. 50.3%; adjusted OR, 3.75; 95% CI, 2.23–6.32; *p* < 0.001) was more frequent in the remimazolam group (Table 2).

### 3.4. Association Between Flatus Recovery and Downstream Outcomes

In exploratory analyses, longer time to first flatus was associated with delayed downstream recovery. After adjustment for prespecified clinical covariates, each 10% increase in time to first flatus was associated with higher odds of protocol-defined diet delay (OR, 1.12; 95% CI, 1.04–1.21) and prolonged hospital stay (OR, 1.13; 95% CI, 1.05–1.21). Similar associations were observed after further adjustment for anesthetic maintenance strategy (Supplementary Table S3). This pattern was also broadly consistent with spline-based analysis, which showed a higher adjusted probability of prolonged hospital stay with longer time to first flatus, particularly beyond the median range (Supplementary Figure S1).

### 3.5. Postoperative Inflammatory Markers

Postoperative day 1 inflammatory markers were lower in the remimazolam group. Median POD 1 hs-CRP was 0.55 [0.31–1.25] mg/dL in the remimazolam group and 1.36 [0.59–2.56] mg/dL in the desflurane group. Median POD 1 CAR was 0.13 [0.07–0.31] and 0.35 [0.15–0.63], respectively (Table 2). After adjustment for age, modified CCI ≥ 3, acute cholecystitis, previous abdominal surgery, preoperative ERCP/PTGBD, and preoperative hs-CRP, remimazolam-based maintenance remained associated with lower POD 1 hs-CRP (geometric mean ratio, 0.51; 95% CI, 0.40–0.64; *p* < 0.001) and lower POD 1 CAR (geometric mean ratio, 0.51; 95% CI, 0.41–0.64; *p* < 0.001) (Table 2, Figure 2). Analyses of POD 1 inflammatory markers were restricted to patients with available preoperative and POD 1 laboratory data (remimazolam, *n* = 167; desflurane, *n* = 137).

### 3.6. Later Postoperative Outcomes

Protocol-defined diet delay occurred in 15.2% of patients in the remimazolam group and 20.0% in the desflurane group; after adjustment, this difference did not reach statistical significance (OR, 0.61; 95% CI, 0.32–1.15; *p* = 0.129). Median length of stay was 3.0 [2.0–3.0] days in both groups. Prolonged hospital stay occurred in 24.6% and 23.4% of patients, respectively, and was not associated with anesthetic maintenance strategy after adjustment (OR, 1.06; 95% CI, 0.59–1.89; *p* = 0.845). PONV was similar between groups (13.5% vs. 13.8%), and 30-day readmission was infrequent in both groups (0.6% vs. 2.8%) (Supplementary Table S4).

### 3.7. Sensitivity Analyses

In prespecified sensitivity analyses, the associations of remimazolam-based maintenance with shorter time to first flatus and lower POD 1 inflammatory markers were attenuated after additional adjustment for surgery duration or anesthesia duration but remained statistically significant in models incorporating surgery duration, anesthesia duration, or remifentanil infusion rate (Supplementary Table S2). In additional sensitivity analyses accounting for possible temporal practice variation, the association between remimazolam-based maintenance and shorter time to first flatus remained significant after inclusion of calendar-quarter fixed effects (TR, 0.82; 95% CI, 0.74–0.90; *p* < 0.001). This association was attenuated but remained significant in models further adjusted for calendar-quarter fixed effects and surgery duration (TR, 0.86; 95% CI, 0.78–0.95; *p* = 0.003) or anesthesia duration (TR, 0.87; 95% CI, 0.79–0.95; *p* = 0.003). Similar attenuation was observed for POD 1 inflammatory markers, although the associations remained statistically significant (Supplementary Table S2).

## 4. Discussion

### 4.1. Main Findings

In this study, remimazolam-based maintenance was associated with a distinct early gastrointestinal recovery pattern after standardized laparoscopic cholecystectomy. Time to first flatus was shorter in the remimazolam group by an observed median of 8 h, and this association remained significant after adjustment, corresponding to a 21% shorter recovery time and an adjusted median reduction of approximately 8.0 h. This pattern was also consistent across predefined early recovery windows, with patients in the remimazolam group more likely to achieve first flatus within 24, 30, and 36 h. Remimazolam-based maintenance was additionally associated with lower POD 1 hs-CRP and CAR. In contrast, later postoperative outcomes, including length of stay, PONV, and prolonged hospital stay, were largely similar between groups. These findings suggest that, in this short-stay LC pathway, differences related to anesthetic maintenance strategy were more apparent in early recovery measures than in later discharge-related outcomes.

### 4.2. Interpretation in a Standardized Short-Stay Pathway

This pattern is particularly relevant in laparoscopic cholecystectomy because LC is a low-complication, fast-recovery procedure commonly managed within a structured short-stay pathway [3,4]. In this setting, standardized diet advancement, discharge routines, and low event rates compress variability in downstream outcomes and may limit the sensitivity of conventional postoperative endpoints [5,6,7]. Accordingly, the absence of between-group differences in later outcomes should not be interpreted to mean that postoperative recovery itself was equivalent. Within a fixed pathway, hospital discharge may converge despite biologically and functionally different recovery courses [7,9,10,11,12].

### 4.3. Early Recovery Beyond Discharge-Based Outcomes

These findings are consistent with QoR and ERAS literature, in which postoperative recovery is regarded as multidimensional and not fully captured by discharge timing alone [5,6,9,10,11,12,13,14,15]. Length of stay is affected by institutional routines, discharge logistics, and perioperative pathway design, in addition to physiologic recovery [6,7,9,10,11,12]. This issue is especially relevant in short-stay LC, where a common discharge target can reduce observable differences in later outcomes. In this setting, early functional measures such as time to first flatus may provide information that is not reflected by length of stay alone. Recent meta-analytic evidence from ERAS trials also supports interpreting length of stay and readmission in relation to the pathway in which they are measured [16].

### 4.4. Relevance of Time to First Flatus

Time to first flatus is a simple measure, but in abdominal surgery it reflects return of bowel function and has practical relevance for diet progression [8]. In the present study, longer time to first flatus was associated with higher odds of protocol-defined diet delay and prolonged hospital stay. These exploratory findings suggest that flatus time captured a recovery feature relevant to pathway progression. The adjusted median reduction of approximately 8.0 h should therefore be interpreted not as an isolated clock–time difference but as an earlier shift in gastrointestinal recovery within an otherwise standardized short-stay pathway. Given the low-risk nature and rapid recovery course of laparoscopic cholecystectomy, this difference should be interpreted as an early functional recovery signal rather than as evidence of a major difference in overall clinical outcomes.

### 4.5. Intravenous and Inhalational Maintenance Strategies

The observed difference in time to first flatus can be considered in relation to prior comparisons of intravenous and inhalational maintenance. Studies in laparoscopic surgery, mainly involving propofol-based TIVA, have reported more favorable gastrointestinal recovery with intravenous than with inhalational anesthesia [17,18]. Volatile anesthetic exposure may influence postoperative gastrointestinal recovery through effects on autonomic regulation, smooth muscle activity, inflammatory response, and recovery kinetics [17,18,19]. The present study evaluated remimazolam-based, rather than propofol-based, intravenous maintenance; therefore, the findings should be interpreted as a strategy-level comparison rather than evidence for a specific drug effect. As remimazolam-based and desflurane-based maintenance were delivered as fixed clinical strategies with distinct drug combinations, the present study cannot separate the pharmacologic effect of remimazolam from the broader effect of the intravenous maintenance strategy. Furthermore, the mean remifentanil infusion rate was higher in the remimazolam group, making an explanation based solely on lower intraoperative opioid intensity unlikely [20]. The association may reflect multiple features of the maintenance approach, including hypnotic–opioid balance, recovery kinetics, and differences related to volatile anesthetic exposure [17,18,19]. However, the present study was not designed to identify a specific pharmacologic mechanism linking remimazolam-based maintenance to earlier gastrointestinal recovery. Therefore, the findings should be interpreted as a clinical association between anesthetic maintenance strategy and early recovery trajectory rather than as evidence of a direct pro-motility effect of remimazolam.

### 4.6. Postoperative Inflammatory Findings

The lower POD 1 hs-CRP and CAR in the remimazolam group provide supportive, but not mechanistic, evidence. The direction of these biomarker findings was consistent with the earlier flatus recovery observed in the same group. Anesthetic technique may influence perioperative inflammatory responses, and experimental and clinical studies have suggested interactions among anesthetic exposure, inflammatory signaling, and gastrointestinal recovery [19,21]. However, this study was not designed to determine whether inflammation mediated the association between maintenance strategy and gastrointestinal recovery. A randomized trial in ureteroscopic lithotripsy also reported lower 24-h inflammatory marker changes with remimazolam-based anesthesia [21], but the relevance of that finding to LC remains uncertain. Therefore, the inflammatory results should be interpreted as complementary evidence of early recovery differences rather than proof of a specific biological pathway.

### 4.7. Limitations and Future Directions

Several limitations should be acknowledged. First, this was a retrospective single-center study with nonrandom treatment allocation, and residual confounding cannot be excluded. Because anesthetic care was delivered by a single attending anesthesiologist, provider-level heterogeneity was limited. However, the choice of maintenance strategy was not randomized and may have reflected temporal practice preference, workflow, or other unmeasured clinical factors. Baseline and perioperative characteristics also differed between groups, including age, comorbidity burden, surgery duration, anesthesia duration, and remifentanil infusion rate. We addressed these differences using prespecified multivariable adjustment and robustness analyses incorporating operative exposure, remifentanil infusion rate, and calendar-quarter fixed effects. However, these analyses cannot fully eliminate selection bias or unmeasured confounding. Therefore, the present findings should be interpreted as associations between real-world anesthetic maintenance strategies and early recovery trajectory, rather than as causal evidence for an isolated pharmacologic effect of remimazolam-based anesthesia. Second, because each maintenance strategy consisted of a fixed combination of drugs and technique, the present study cannot distinguish the pharmacologic effect of a specific agent from the broader effect of the anesthetic maintenance strategy. The remimazolam group had a higher comorbidity burden but shorter operative and anesthetic exposure. Although robustness analyses incorporating surgery duration, anesthesia duration, and remifentanil infusion rate showed that the main associations persisted, estimates were attenuated after adjustment for operative exposure. These findings should therefore be interpreted as strategy-level associations rather than isolated drug effects. Third, we did not assess formal QoR instruments, patient-reported recovery, mobilization, or other broader functional recovery domains; thus, time to first flatus does not capture the entirety of postoperative recovery. Fourth, time to first flatus was based on routine nursing documentation and may therefore be subject to measurement imprecision, although this outcome was assessed as part of standard postoperative care in both groups. Nevertheless, the direction of the findings was consistent across gastrointestinal recovery, threshold-based analyses, inflammatory markers, and sensitivity analyses. Future prospective studies incorporating QoR instruments and broader functional recovery measures are needed to better define the clinical relevance of these differences.

## 5. Conclusions

In standardized laparoscopic cholecystectomy, remimazolam-based maintenance was associated with earlier gastrointestinal recovery and lower POD 1 inflammatory markers, whereas later discharge-based outcomes were largely similar between groups. These findings support the use of early functional recovery measures, in addition to discharge-based outcomes, when evaluating perioperative recovery in low-event short-stay surgery.

## Figures and Tables

**Table 1:** Baseline and perioperative characteristics according to anesthetic maintenance strategy.

Variable	Remimazolam (*n* = 171)	Desflurane (*n* = 145)	*p* Value
Baseline characteristics			
Age, yr	63.0 [53.5–71.5]	60.0 [48.0–69.0]	0.033
Female sex	99 (57.9)	78 (53.8)	0.496
BMI, kg/m^2^	24.4 [22.2–26.9]	24.6 [22.0–27.3]	0.464
ASA physical status ≥ III	73 (42.7)	46 (31.7)	0.048
Modified CCI ≥ 3	38 (22.2)	13 (9.0)	0.002
Previous abdominal surgery	62 (36.3)	54 (37.2)	0.907
Acute cholecystitis	19 (11.1)	18 (12.4)	0.729
ERCP and/or PTGBD before surgery	21 (12.3)	20 (13.8)	0.738
Preoperative hs-CRP, mg/dL	0.07 [0.04–0.15]	0.07 [0.04–0.16]	0.612
Perioperative characteristics			
Surgery duration, min	30 [25–45]	45 [35–60]	<0.001
Anesthesia duration, min	50 [45–65]	60 [50–80]	<0.001
Remifentanil infusion rate, μg/kg/min	0.108 [0.091–0.126]	0.089 [0.075–0.101]	<0.001
PCA use	166 (97.1)	139 (95.9)	0.760

Values are presented as median [interquartile range] or number (%). ASA, American Society of Anesthesiologists; CCI, Charlson comorbidity index; ERCP, endoscopic retrograde cholangiopancreatography; PTGBD, percutaneous transhepatic gallbladder drainage; hs-CRP, high-sensitivity C-reactive protein; PCA, patient-controlled analgesia.

**Table 2:** Postoperative recovery outcomes and adjusted associations according to anesthetic maintenance strategy.

Outcome	Remimazolam	Desflurane	Adjusted Estimate (95% CI)	Adjusted *p* Value
Early gastrointestinal recovery				
Time to first flatus, h	28.0 [24.0–37.0]	36.0 [28.0–52.0]	Time ratio 0.79 (0.72–0.86)	<0.001
Flatus within 24 h	47 (27.5)	19 (13.1)	OR 2.42 (1.33–4.41)	0.004
Flatus within 30 h	100 (58.5)	58 (40.0)	OR 2.47 (1.53–3.99)	<0.001
Flatus within 36 h	128 (74.9)	73 (50.3)	OR 3.75 (2.23–6.32)	<0.001
Postoperative inflammatory burden				
POD 1 hs-CRP, mg/dL *	0.55 [0.31–1.25]	1.36 [0.59–2.56]	GMR 0.51 (0.40–0.64)	<0.001
POD 1 CAR *	0.13 [0.07–0.31]	0.35 [0.15–0.63]	GMR 0.51 (0.41–0.64)	<0.001
Later postoperative outcomes				
Protocol-defined diet delay	26 (15.2)	29 (20.0)	OR 0.61 (0.32–1.15)	0.129
Length of stay, d	3.0 [2.0–3.0]	3.0 [2.0–3.0]	—	—
Prolonged hospital stay	42 (24.6)	34 (23.4)	OR 1.06 (0.59–1.89)	0.845

Values are presented as median [interquartile range] or number (%), unless otherwise indicated. Adjusted estimates were derived from multivariable models including age (per 10 years), modified CCI ≥ 3, acute cholecystitis, previous abdominal surgery, and preoperative biliary intervention (ERCP and/or PTGBD). Models for postoperative day 1 inflammatory markers were additionally adjusted for preoperative hs-CRP; because a small number of hs-CRP and CAR values were zero, 0.01 was added before logarithmic transformation. A time ratio < 1 indicates faster gastrointestinal recovery in the remimazolam group. Model-derived adjusted median times to first flatus were 30.3 h in the remimazolam group and 38.3 h in the desflurane group, corresponding to an adjusted median reduction of approximately 8.0 h. * POD 1 inflammatory marker analyses were performed in patients with available preoperative and POD 1 laboratory data (remimazolam *n* = 167; desflurane *n* = 137). Exponentiated coefficients from log-transformed models are presented as geometric mean ratios (GMRs). CAR, C-reactive protein-to-albumin ratio; CI, confidence interval; hs-CRP, high-sensitivity C-reactive protein; LOS, length of stay; POD 1, postoperative day 1.

## Data Availability

The data presented in this study are available on request from the corresponding author. The data are not publicly available due to privacy and ethical restrictions.

## Supplementary Materials

The following supporting information can be downloaded at: https://www.mdpi.com/article/10.3390/jcm15114202/s1: Table S1: Full multivariable model coefficients for primary and key secondary outcomes; Table S2: Sensitivity analyses for the association of remimazolam-based maintenance with key outcomes; Table S3: Association of time to first flatus with downstream recovery outcomes; Table S4: Additional postoperative outcomes according to anesthetic maintenance strategy; Figure S1: Adjusted probability of prolonged hospital stay according to time to first flatus.
