# Supplementary material for: Association of Remimazolam-Based Versus Desflurane-Based Maintenance with Early Gastrointestinal Recovery After Laparoscopic Cholecystectomy: A Single-Center Retrospective Cohort Study
Source: J Clin Med. 2026 May 29;15(11):4202. doi: 10.3390/jcm15114202 (PMC13258766; doi:10.3390/jcm15114202)
Supplement: Supplementary file 1 [file jcm-15-04202-s001.zip › Supplementary Table S1_.pdf]

Supplementary Table S1. Full multivariable model coefficients for primary and key secondary outcomes

Outcome	Measure	Variable	Adjusted estimate (95% CI)	P value
Time to first flatus	Time ratio	Remimazolam-based maintenance	0.79 (0.72–0.86)	< 0.001
		Age (per 10 years)	1.05 (1.02–1.09)	0.004
		Modified CCI ≥ 3	1.02 (0.91–1.16)	0.704
		Acute cholecystitis	1.02 (0.87–1.19)	0.784
		Previous abdominal surgery	0.96 (0.88–1.05)	0.414
		ERCP and/or PTGBD before surgery	1.00 (0.86–1.17)	0.970
POD 1 hs-CRP	GMR	Remimazolam-based maintenance	0.51 (0.40–0.64)	< 0.001
		Age (per 10 years)	1.16 (1.05–1.27)	0.002
		Modified CCI ≥ 3	1.16 (0.84–1.62)	0.370
		Acute cholecystitis	1.29 (0.84–1.98)	0.237
		Previous abdominal surgery	0.98 (0.77–1.25)	0.898
		ERCP and/or PTGBD before surgery	1.40 (0.92–2.12)	0.116
		Preoperative hs-CRP (log-transformed)	1.36 (1.23–1.51)	< 0.001
POD 1 CAR	GMR	Remimazolam-based maintenance	0.51 (0.41–0.64)	< 0.001
		Age (per 10 years)	1.16 (1.06–1.26)	0.001
		Modified CCI ≥ 3	1.17 (0.86–1.58)	0.317
		Acute cholecystitis	1.37 (0.92–2.03)	0.117
		Previous abdominal surgery	0.97 (0.78–1.22)	0.812
		ERCP and/or PTGBD before surgery	1.38 (0.94–2.03)	0.099
		Preoperative hs-CRP (log-transformed)	1.34 (1.22–1.48)	< 0.001
Diet delay	Odds ratio	Remimazolam-based maintenance	0.61 (0.32–1.15)	0.129

Outcome	Measure	Variable	Adjusted estimate (95% CI)	P value
		Age (per 10 years)	1.71 (1.29–2.27)	< 0.001
		Modified CCI ≥ 3	0.78 (0.33–1.88)	0.587
		Acute cholecystitis	2.00 (0.78–5.10)	0.148
		Previous abdominal surgery	0.79 (0.41–1.53)	0.480
		ERCP and/or PTGBD before surgery	1.75 (0.71–4.35)	0.227
Prolonged hospital stay	Odds ratio	Remimazolam-based maintenance	1.06 (0.59–1.89)	0.845
		Age (per 10 years)	1.26 (1.00–1.60)	0.049
		Modified CCI ≥ 3	0.95 (0.44–2.09)	0.905
		Acute cholecystitis	3.62 (1.54–8.50)	0.003
		Previous abdominal surgery	0.79 (0.43–1.44)	0.438
		ERCP and/or PTGBD before surgery	3.48 (1.54–7.86)	0.003

Adjusted estimates are presented as time ratios for time to first flatus, geometric mean ratios (GMRs) for POD 1 hs-CRP and POD 1 CAR, and odds ratios for binary outcomes. All models included age (per 10 years), modified CCI ≥3, acute cholecystitis, previous abdominal surgery, and preoperative biliary intervention (ERCP and/or PTGBD). POD 1 inflammatory marker models were additionally adjusted for preoperative hs-CRP; because a small number of hs-CRP and CAR values were zero, 0.01 was added before logarithmic transformation. Exponentiated coefficients from log-transformed models are presented as GMRs. CAR, C-reactive protein-to-albumin ratio; hs-CRP, high-sensitivity C-reactive protein; POD 1, postoperative day 1.
