# Supplementary material for: Association of Remimazolam-Based Versus Desflurane-Based Maintenance with Early Gastrointestinal Recovery After Laparoscopic Cholecystectomy: A Single-Center Retrospective Cohort Study
Source: J Clin Med. 2026 May 29;15(11):4202. doi: 10.3390/jcm15114202 (PMC13258766; doi:10.3390/jcm15114202)
Supplement: Supplementary file 1 [file jcm-15-04202-s001.zip › Supplementary Table S2_.pdf]

Supplementary Table S2. Sensitivity analyses for the association of remimazolam-based maintenance with key outcomes

Outcome	Sensitivity model	Adjusted estimate (95% CI)	P value
Time to first flatus	Main model	0.79 (0.72–0.86)	< 0.001
	Additional adjustment for surgery duration	0.86 (0.78–0.94)	< 0.001
	Additional adjustment for anesthesia duration	0.86 (0.79–0.94)	0.001
	Additional adjustment for remifentanil infusion rate	0.79 (0.72–0.87)	< 0.001
	Additional adjustment for calendar-quarter fixed effects	0.82 (0.74–0.90)	< 0.001
	Additional adjustment for calendar-quarter fixed effects and surgery duration	0.86 (0.78–0.95)	0.003
	Additional adjustment for calendar-quarter fixed effects and anesthesia duration	0.87 (0.79–0.95)	0.003
POD 1 hs-CRP	Main model	0.51 (0.40–0.64)	< 0.001
	Additional adjustment for surgery duration	0.63 (0.50–0.80)	< 0.001
	Additional adjustment for anesthesia duration	0.63 (0.50–0.80)	< 0.001
	Additional adjustment for remifentanil infusion rate	0.55 (0.43–0.71)	< 0.001
	Additional adjustment for calendar-quarter fixed effects	0.57 (0.44–0.74)	< 0.001
	Additional adjustment for calendar-quarter fixed effects and surgery duration	0.67 (0.52–0.86)	0.002
POD 1 CAR	Main model	0.51 (0.41–0.64)	< 0.001
	Additional adjustment for surgery duration	0.64 (0.51–0.79)	< 0.001
	Additional adjustment for anesthesia duration	0.63 (0.51–0.79)	< 0.001
	Additional adjustment for remifentanil infusion rate	0.55 (0.44–0.69)	< 0.001
	Additional adjustment for calendar-quarter fixed effects	0.57 (0.45–0.73)	< 0.001

Outcome	Sensitivity model	Adjusted estimate (95% CI)	P value
	Additional adjustment for calendar-quarter fixed effects and surgery duration	0.67 (0.53–0.85)	< 0.001

The main model included age (per 10 years), modified CCI ≥3, acute cholecystitis, previous abdominal surgery, and preoperative biliary intervention (ERCP and/or PTGBD). Robustness analyses additionally adjusted for surgery duration, anesthesia duration, or mean weight-normalized intraoperative remifentanil infusion rate. POD 1 inflammatory marker models were additionally adjusted for preoperative hs-CRP; because a small number of hs-CRP and CAR values were zero, 0.01 was added before logarithmic transformation. Results for time to first flatus are presented as time ratios, and results for POD 1 hs-CRP and POD 1 CAR are presented as geometric mean ratios (GMRs). Additional sensitivity analyses were performed to account for possible temporal practice variation by including calendar-quarter fixed effects. For the primary outcome, further sensitivity models combined calendar-quarter fixed effects with surgery duration or anesthesia duration. ERCP, endoscopic retrograde cholangiopancreatography; PTGBD, percutaneous transhepatic gallbladder drainage; hs-CRP, high-sensitivity C-reactive protein.
