# Supplementary material for: Association of Remimazolam-Based Versus Desflurane-Based Maintenance with Early Gastrointestinal Recovery After Laparoscopic Cholecystectomy: A Single-Center Retrospective Cohort Study
Source: J Clin Med. 2026 May 29;15(11):4202. doi: 10.3390/jcm15114202 (PMC13258766; doi:10.3390/jcm15114202)
Supplement: Supplementary file 1 [file jcm-15-04202-s001.zip › Supplementary Table S3_.pdf]

Supplementary Table S3. Association of time to first flatus with downstream recovery outcomes

Outcome	Model	Odds ratio (95% CI)	P value
Protocol-defined diet delay	Adjusted for clinical covariates	1.12 (1.04–1.21)	0.003
Protocol-defined diet delay	Further adjusted for anesthetic maintenance strategy	1.11 (1.03–1.20)	0.009
Prolonged hospital stay	Adjusted for clinical covariates	1.13 (1.05–1.21)	< 0.001
Prolonged hospital stay	Further adjusted for anesthetic maintenance strategy	1.14 (1.06–1.23)	< 0.001

Odds ratios represent the change in risk associated with each 10% increase in time to first flatus. Clinical covariates included age (per 10 years), modified CCI ≥3, acute cholecystitis, previous abdominal surgery, and preoperative biliary intervention (ERCP and/or PTGBD). Additional models were further adjusted for anesthetic maintenance strategy. LOS, length of stay.
