# Supplementary material for: Association of Remimazolam-Based Versus Desflurane-Based Maintenance with Early Gastrointestinal Recovery After Laparoscopic Cholecystectomy: A Single-Center Retrospective Cohort Study
Source: J Clin Med. 2026 May 29;15(11):4202. doi: 10.3390/jcm15114202 (PMC13258766; doi:10.3390/jcm15114202)
Supplement: Supplementary file 1 [file jcm-15-04202-s001.zip › Supplementary Table S4_.pdf]

Supplementary Table S4. Additional postoperative outcomes according to anesthetic maintenance strategy

Outcome	Remimazolam (n = 171)	Desflurane (n = 145)	P value
PONV	23 (13.5)	20 (13.8)	1.000
30-day readmission	1 (0.6)	4 (2.8)	—

Values are presented as number (%). P values, where shown, were derived from Fisher's exact test. Thirty-day readmission was infrequent and is therefore presented descriptively only. PONV, postoperative nausea and vomiting.
