# Supplementary figures and images for: Association of Remimazolam-Based Versus Desflurane-Based Maintenance with Early Gastrointestinal Recovery After Laparoscopic Cholecystectomy: A Single-Center Retrospective Cohort Study
Source: J Clin Med. 2026 May 29;15(11):4202. doi: 10.3390/jcm15114202 (PMC13258766; doi:10.3390/jcm15114202)

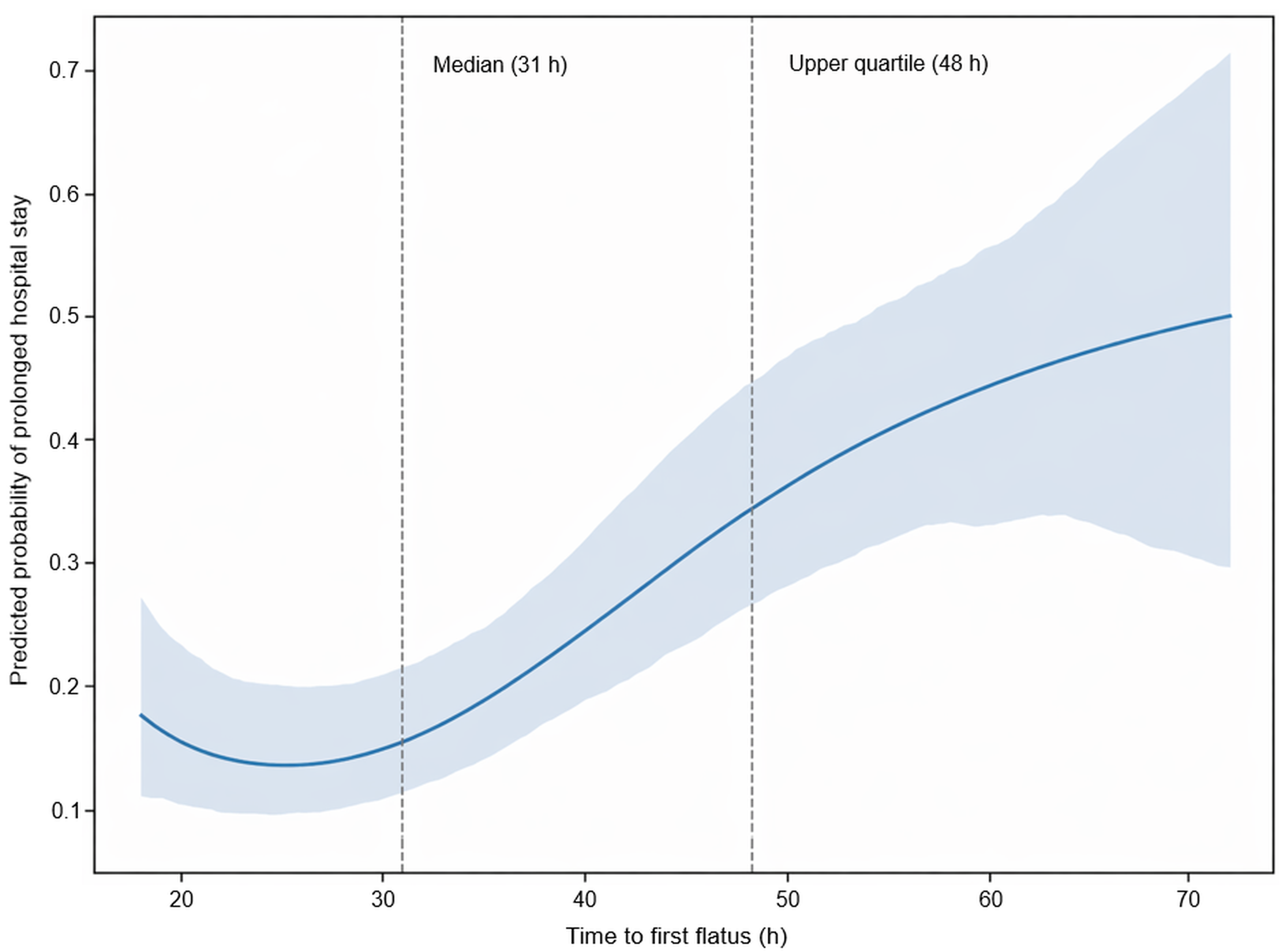

Supplement: Supplementary file 1 [file jcm-15-04202-s001.zip › Figure_S1_.png]
